# Innovative new model predicts glucose levels without poking or prodding

**DOI:** 10.1038/s41746-021-00501-9

**Published:** 2021-08-20

**Authors:** Leia Wedlund, Joseph Kvedar

**Affiliations:** 1 Harvard Medical School, Boston, MA USA; 2 Mass General Brigham, Boston, MA USA

**Keywords:** Diagnostic markers, Metabolic diseases

## Abstract

With the prevalence of type II diabetes rising rapidly it has become increasingly apparent that something must be done to stem the tide. While pharmaceutical treatments aimed at lowering average blood sugar are an important tool in this endeavor, it is equally (if not more) important to motivate patients to make healthy diet and exercise choices. Recent advances in non-invasive glucose monitoring suggest that real-time patient feedback may soon be available to help guide daily patient decision-making.

As the prevalence of type II diabetes rises, hospitalization costs skyrocket, and kidney transplant waitlists lengthen^1,2^, physicians and researchers have turned their attention to stemming the flow by preventing progression from “prediabetes” to full-blown type II diabetes.

One critical part of this effort is motivating and enabling patients to make healthier lifestyle choices. Through diet and exercise, people with prediabetes can halve their risk of progression to type 2 diabetes^3^, and research shows that patients are motivated to make better diet/exercise decisions when provided with their exact glucose values^4^. However, glucose monitoring, whether accomplished via continuous glucose monitor (CGM) or regular fingersticks, has always been an invasive process. Even amongst patients with a diagnosis of type I or type II diabetes, only 63% report checking their glucose levels at least once per day, and glucose monitoring is significantly less common in the prediabetes population^5^. This creates an enormous unmet clinical need for noninvasive glucose monitoring tools.

Enter, the Duke researchers behind a novel machine learning model which predicts interstitial glucose levels using non-invasive measurements with impressive accuracy. Based on existing evidence that physiologic parameters such as heart rate can reflect glucose fluctuations^6^ they developed a model combining 69 inputs to predict glucose levels in 16 study participants with diagnosed prediabetes or high-normal blood glucose levels. The model inputs included demographic/historical data such as biological sex, food log (recorded by study participant), and last HgbA1c measurement, as well as biomarkers of stress, activity, and circadian rhythm. These biomarkers were collected from a smartwatch that captured heart rate variability, skin temperature, sweating (electrodermal activity), and accelerometry^7^.

To train and validate this non-invasive glucose prediction model, all participants were outfitted with a Dexcom G6 continuous glucose monitor for the duration of the study, providing a benchmark for true interstitial glucose levels. As the machine learning model was “learning”, it used the Dexcom benchmark to determine the amount of weight that should be placed on each non-invasive data point (heart rate, food journal entries, etc.) to predict glucose levels with the highest possible accuracy. Once the training period was complete, for the remainder of the study period, the model predicted glucose levels using *only* non-invasive data points and these predictions were checked against the Dexcom benchmark to judge overall accuracy. Ultimately, this population model was able to predict exact interstitial glucose levels for study participants using purely non-invasive data with a stunning 87% accuracy^7^. This degree of accuracy is in line with that of today’s interstitial glucose monitors as compared to blood glucose measurements^8^, suggesting that this model could one-day be used in lieu of continuous glucose monitoring for some individuals.

With this important step forward, we move closer to the goal of non-invasive glucose monitoring and less burdensome, higher quality care. This type of non-invasive glucose monitoring system could help motivate prediabetes patients to make important diet and exercise changes. However, the full benefit of this technology can only be realized with careful attention to accessibility and ease-of-use, two frequent stumbling blocks in the transition from research to clinical use.

The biomarkers used in this paper were collected from the Empatica E4 smartwatch, a device that costs over $1500; and while the research participants in this study dutifully recorded food journals each day, manual food journals are notoriously burdensome. As researchers consider scaling this work and eventually introducing it in a clinical setting, these data-collection modalities will likely require modifications to improve affordability and ease-of-use. For instance, in a future iteration, the model could be adjusted to rely upon biomarkers collected by more commonly available commercial fitness trackers. The food journal element also presents opportunities for innovation and modification. In the near-term, it is conceivable that this patient burden could be reduced by the use of verbal food diaries interpreted by automated voice recognition systems. In the longer-term, patient burden could be all but eliminated by computer vision technologies (currently under development) that recognize and classify all elements of a meal from a single picture^9,10^.

Non-invasive glucose monitoring once seemed like an impossible challenge, but today’s technology and the incredible work of Bent et al. promise a future in which daily fingersticks are obsolete. The new challenge will be realizing that promise in an accessible and practical way that benefits all patients.
